# Differential Effects of Abdominal Bracing and Hollowing on Scapular Muscle Activation Patterns During the Y-Raise Exercise

**DOI:** 10.3390/jcm15187282

**Published:** 2026-09-19

**Authors:** Jihwan Song, Ungbeom Kim, Haneul Lee

**Affiliations:** 1 Graduate School, Department of Physical Therapy, College of Health Science, Gachon University, Incheon 21936, Republic of Korea; songjh0921@naver.com; 2 Department of Physical Therapy, College of Health Science, Gachon University, Incheon 21936, Republic of Korea; 5chungi@gachon.ac.kr

**Keywords:** electromyography, muscle activation, scapular muscle activation, rounded shoulder posture

## Abstract

**Background:** Trunk stability is closely linked to scapular muscle recruitment during upper-limb activities, yet the differential effects of abdominal bracing and hollowing on scapular muscle activation patterns remain unclear. **Objective:** To compare the effects of abdominal bracing, hollowing, and normal breathing on scapular muscle activation and activation ratios during the Y-raise exercise in individuals with rounded shoulder posture (RSP). **Methods:** Thirty-six individuals with RSP participated in this within-subject repeated-measures study. Surface EMG was used to record upper trapezius (UT), lower trapezius (LT), and serratus anterior (SA) activity during the Y-raise exercise under three conditions: normal breathing, abdominal hollowing, and abdominal bracing. Muscle activation (%MVIC) and UT/LT and UT/SA ratios were compared using repeated-measures ANOVA with Bonferroni-adjusted pairwise comparisons. **Results:** The bracing instruction condition was associated with greater LT activation than hollowing and normal breathing on both sides (adjusted *p* < 0.05), whereas UT activation did not differ across conditions. Both bracing and hollowing were associated with greater SA activation than normal breathing on both sides (adjusted *p* < 0.05). Bracing was associated with lower UT/LT ratios than both hollowing and normal breathing on both sides. The UT/SA ratio was lower with bracing than with normal breathing on both sides and lower than with hollowing only on the left side. **Conclusions:** Abdominal bracing instruction was associated with acute changes in relative scapular EMG activation during the Y-raise, characterized primarily by greater LT activation rather than reduced UT activity. These preliminary findings reflect responses to different instructional conditions, and their clinical relevance in symptomatic populations remains to be determined.

## 1. Introduction

Shoulder pain is common worldwide, with a median community prevalence of approximately 16%, and shoulder disorders impose substantial societal costs through healthcare use, work absence, and productivity loss [1,2]. Accordingly, approaches intended to reduce modifiable shoulder muscle demands have received increasing attention, including wearable support during occupational tasks [3]. Exercise-based strategies aimed at optimizing trunk–scapular control may provide a complementary and clinically accessible approach, although their effects on shoulder disorder prevention and healthcare costs remain to be established.

Core stability plays a fundamental role in maintaining postural control, efficient load transfer, and functional movement throughout the kinetic chain. Postural control depends on coordinated neuromuscular responses across multiple body segments, and muscle strength and neuromuscular coordination contribute to postural stability during functional tasks [4]. Within this broader kinetic-chain framework, a stable trunk provides the mechanical and neuromuscular foundation necessary for efficient upper-limb movement, particularly during tasks requiring coordinated shoulder movement [5,6]. When trunk stability is insufficient, the efficiency of force transmission between the spine and the shoulder complex may be compromised, potentially contributing to compensatory overactivation of the upper trapezius (UT) and insufficient activation of the lower trapezius (LT) and serratus anterior (SA). Such neuromuscular imbalances can impair scapular upward rotation, alter shoulder kinematics, and increase the risk of overuse-related shoulder disorders [7,8,9].

Recent evidence suggests that trunk stabilization is closely linked to scapular muscle recruitment during upper-limb activities. Pre-activation of core musculature has been shown to improve scapular alignment, reduce excessive UT activity, and enhance LT and SA engagement [10,11]. These findings support the concept that the trunk and shoulder complex function as an integrated neuromuscular unit, wherein proximal stability serves as a prerequisite for efficient distal motor control.

To enhance trunk stability, two primary motor control strategies have been widely utilized: abdominal hollowing and abdominal bracing. Abdominal hollowing selectively recruits deep local stabilizers, particularly the transversus abdominis and multifidus, promoting segmental spinal control [12]. In contrast, abdominal bracing involves co-contraction of both deep and superficial trunk muscles, including the obliques and rectus abdominis, resulting in increased intra-abdominal pressure and enhanced global trunk stiffness [13,14]. Although both strategies aim to improve lumbopelvic stability, they differ fundamentally in their neuromuscular activation patterns and may therefore exert distinct effects on force transmission and inter-muscular coordination across the kinetic chain.

The Y-raise exercise, which promotes activation of the LT and SA while minimizing UT dominance, provides a controlled and functionally relevant context for evaluating scapular upward rotation [9]. Examining how different core activation strategies modulate scapular muscle recruitment during this task may provide important insight into the neuromechanical relationship between trunk stabilization and shoulder function.

Despite growing evidence supporting the role of trunk stabilization in upper-limb function, the specific effects of different core activation strategies on scapular muscle activation during functional movements remain unclear. Previous studies have primarily examined core stabilization and scapular muscle activity in isolation, and few have directly compared how abdominal hollowing and bracing differentially influence scapular muscle activation during functional upper-limb tasks [15,16]. Moreover, prior research has largely focused on individual muscle activation levels, with limited attention given to muscle activation ratios, which reflect the relative contributions of synergistic muscles and may provide additional information about relative scapular EMG activation patterns. Therefore, individuals with rounded shoulder posture (RSP) may provide an informative model for examining how different abdominal activation strategies influence scapular muscle activation during upper-limb exercise [17,18].

Therefore, the purpose of this study was to compare the electromyographic (EMG) activity of the UT, LT, and SA muscles during Y-raise exercises performed under three abdominal conditions: normal breathing, abdominal hollowing, and abdominal bracing. It was hypothesized that abdominal bracing would produce greater LT and SA activation and lower UT/LT and UT/SA ratios compared with abdominal hollowing and normal breathing.

## 2. Materials and Methods

### 2.1. Study Design and Ethical Considerations

The experimental framework comprised five sequential stages: participant screening, familiarization with the abdominal activation strategies, randomized performance of the Y-raise under three conditions, EMG data collection, and comparison of scapular muscle activation and activation ratios across conditions.

This study was designed as a single-session, laboratory-based, within-subject repeated-measures experimental study to investigate the acute effects of different abdominal activation strategies on scapular muscle activity during the Y-raise exercise.

The study protocol adhered to the principles of the Declaration of Helsinki and was approved by the Institutional Review Board of Gachon University (IRB No.1044396-202501-HR-009-01). Prior to participation, all individuals received a detailed explanation of the study procedures and provided written informed consent.

### 2.2. Participants

Thirty-six healthy adults with RSP characteristics participated in this study. Participants were recruited through advertisements at Gachon University. The inclusion criteria were as follows: (1) age ≥ 18 years and (2) a sagittal shoulder-C7 angle < 52°, consistent with the operational definition of RSP. The exclusion criteria included: (1) a history of musculoskeletal or neurological disorders affecting the spine or shoulder, and (2) any history of spinal or shoulder surgery within the previous year. RSP was operationally defined as a sagittal shoulder-C7 angle < 52°, in accordance with previously reported photogrammetric criteria [19,20]. This criterion was used solely to determine eligibility and characterize postural alignment rather than to diagnose clinical shoulder disorder. The shoulder angle was used only as a static screening measure and was not analyzed as a study outcome. Photographic assessment of sagittal shoulder posture has demonstrated acceptable reliability and validity [20]. Smartphone-based photography combined with ImageJ (version 1.54g; National Institutes of Health, Bethesda, MD, USA) was used as a simple and noninvasive screening approach. To improve measurement consistency, camera distance and height, participant position, and marker placement were standardized. Reflective markers were placed on the C7 spinous process and acromion. Participants stood in a relaxed upright posture with their arms resting naturally at their sides and feet shoulder-width apart. A digital camera (iPhone 12 Pro Max, Apple Inc., Cupertino, CA, USA) was positioned 1 m from the participant at shoulder height [20]. One standardized sagittal-plane image was obtained for each side. The shoulder angle was defined as the angle between a horizontal line passing through C7 and a line connecting C7 to the acromion; a smaller angle indicated a more anterior shoulder position. Each shoulder angle was measured once by the same examiner using ImageJ software (National Institutes of Health, Bethesda, MD, USA) [21].

The required sample size was calculated using G*Power software (version 3.1.9.4; Heinrich-Heine-University, Düsseldorf, Germany). Because prior studies directly comparing abdominal bracing and hollowing during the Y-raise exercise were limited, an a priori power analysis for this exploratory laboratory study was performed using a medium effect size (Cohen’s f = 0.25). The calculation was based on a repeated-measures ANOVA with three within-subject conditions (bracing, hollowing, and normal breathing), a significance level of α = 0.05, and a statistical power of 0.80. The minimum required sample size was 28 participants. Considering a potential dropout rate, 36 participants were recruited.

### 2.3. Experimental Procedures

All experimental procedures were conducted under the supervision of an experienced physical therapist. Participants were first familiarized with abdominal hollowing and bracing techniques, as these strategies may not be intuitive. A 5 min practice session was provided to familiarize participants with the instructions and promote consistent performance [22].

The experimental task consisted of a Y-raise exercise performed under three different conditions: normal breathing, abdominal hollowing, and abdominal bracing. An independent researcher prepared six opaque envelopes representing the six possible condition orders. Each participant selected one envelope before testing, and the envelope was returned before the next selection. Thus, the orders were randomized with replacement and were not intentionally balanced. Each condition consisted of three repetitions for three sets. A rest period of 15 min was provided between conditions to minimize the effects of muscle fatigue [3,23]. During the Y-raise, participants stood 120 cm from an anchor point positioned at chest height while holding a [color] elastic resistance band (TheraBand, Hygenic Corporation, Akron, OH, USA). The same resistance level was used for all participants and was not individualized according to body size or shoulder strength. Band elongation and actual resistance during the exercise were not quantified. From the starting position, participants elevated both arms in the scapular plane to approximately 135°, forming a Y-shape, and then returned to the starting position in a controlled manner. Participants were instructed to exhale during arm elevation and inhale during arm lowering to maintain a consistent breathing pattern across conditions [22].

To differentiate the experimental conditions, specific instructions were provided for each abdominal activation strategy. In the normal breathing condition, participants performed the Y-raise without intentional abdominal contraction. In the abdominal hollowing condition, participants were instructed to gently draw the navel inward toward the spine during exhalation. During familiarization, the examiner palpated the area medial to the anterior superior iliac spine to check performance [24,25]. In the abdominal bracing condition, participants were instructed to contract the entire abdominal wall as if preparing for an external perturbation. Tactile feedback was similarly provided during familiarization by placing a hand on the abdominal wall to check the contraction. During EMG data collection, no manual contact was provided, and abdominal activation was not objectively monitored; participants were instructed to maintain the assigned abdominal strategy throughout each repetition. Accordingly, the experimental conditions were defined by the standardized instructions provided rather than by objectively confirmed differences in abdominal muscle recruitment, intra-abdominal pressure, or trunk stiffness.

### 2.4. Outcome Measures

Surface electromyography (EMG) was used to assess the muscle activity of the LT, UT, and SA. A wireless EMG system (Noraxon DTS, Noraxon USA. Inc., Scottsdale, AZ, USA) was used with a sampling rate of 1000 Hz. Prior to electrode placement, the skin was shaved and cleaned with alcohol to reduce impedance. Pre-gelled bipolar Ag/AgCl disposable electrodes were used, with an inter-electrode distance of 20 mm. Electrodes were placed parallel to the muscle fibers over the muscle belly of each target muscle. Electrodes were positioned on the LT, UT, and SA to record muscle activity during scapular upward rotation tasks.

Maximum voluntary isometric contractions (MVICs) were measured to normalize EMG data, and all values were expressed as a percentage of MVIC (%MVIC). Each muscle was tested in standardized positions based on previous literature. For the LT, MVIC was assessed in a seated position with the shoulder positioned at 135° of abduction and 150° of flexion [26]. To minimize compensatory lower limb contribution, participants were instructed not to push through their feet. Resistance was applied to both arms by the examiner from behind to elicit maximal contraction. For the UT, MVIC was measured in a seated position with the arms relaxed at the sides while performing a shoulder elevation (shrug) task [27]. The examiner applied downward resistance over the shoulders to induce maximal contraction. For the SA, MVIC was measured in a standing position while holding a resistance band with the shoulder elevated above 120° of abduction, and scapular protraction was performed [28]. Resistance was applied horizontally by pulling the band backward to induce maximal contraction. Each MVIC trial was performed three times, and the average value was used for normalization.

Following MVIC assessment, participants performed the Y-raise exercise under three conditions: normal breathing, abdominal hollowing, and abdominal bracing. Each condition was performed three times, and EMG data were collected to assess the activity of scapular upward rotator muscles. The acquired EMG signals were band-pass filtered (20–350 Hz) to reduce low-frequency motion artifacts and high-frequency noise, full-wave rectified, and then smoothed using a 50 ms moving root mean square (RMS) window to obtain the EMG amplitude envelope [29]. For each trial, a stable 3 s segment from the middle phase of the movement was selected for analysis to minimize variability. The mean value across three repetitions was calculated and expressed as a % MVIC.

To characterize relative scapular EMG activation, the UT/LT and UT/SA ratios were calculated. These ratios represent the relative normalized EMG amplitudes between the selected muscles during movement [30]. The UT/LT ratio was calculated by dividing the normalized EMG activity (%MVIC) of the upper trapezius by that of the lower trapezius, and the UT/SA ratio was calculated by dividing the normalized EMG activity (%MVIC) of the upper trapezius by that of the serratus anterior [30].

### 2.5. Statistical Analysis

All statistical analyses were performed using IBM SPSS Statistics (version 26.0; IBM Corp., Armonk, NY, USA). Continuous variables were presented as mean ± standard deviation, and categorical variables were expressed as frequencies. Normality was assessed using the Shapiro–Wilk test, and all outcome variables satisfied the normality assumption (*p* > 0.05). A repeated-measures analysis of variance (ANOVA) was conducted to examine differences in muscle activity (%MVIC) and muscle activation ratios (UT/LT and UT/SA) across the three conditions: normal breathing, abdominal hollowing, and abdominal bracing. Sphericity was evaluated using Mauchly’s test, and the Greenhouse–Geisser correction was applied when sphericity was violated. Significant condition effects were followed by Bonferroni-adjusted pairwise comparisons. Effect sizes for the omnibus analyses were reported as partial eta squared (ηp^2^). For pairwise comparisons, Bonferroni-adjusted mean differences with 95% CIs, adjusted *p*-values, and paired-samples Cohen’s *d*z values with 95% CIs were reported. Statistical significance was set at α = 0.05.

## 3. Results

A total of 40 participants were initially recruited for this study. Of these, three participants withdrew, and one was excluded due to EMG data not meeting the inclusion criteria. Consequently, 36 participants were included in the final analysis. The general characteristics of participants are described in Table 1.

**Figure 1:**
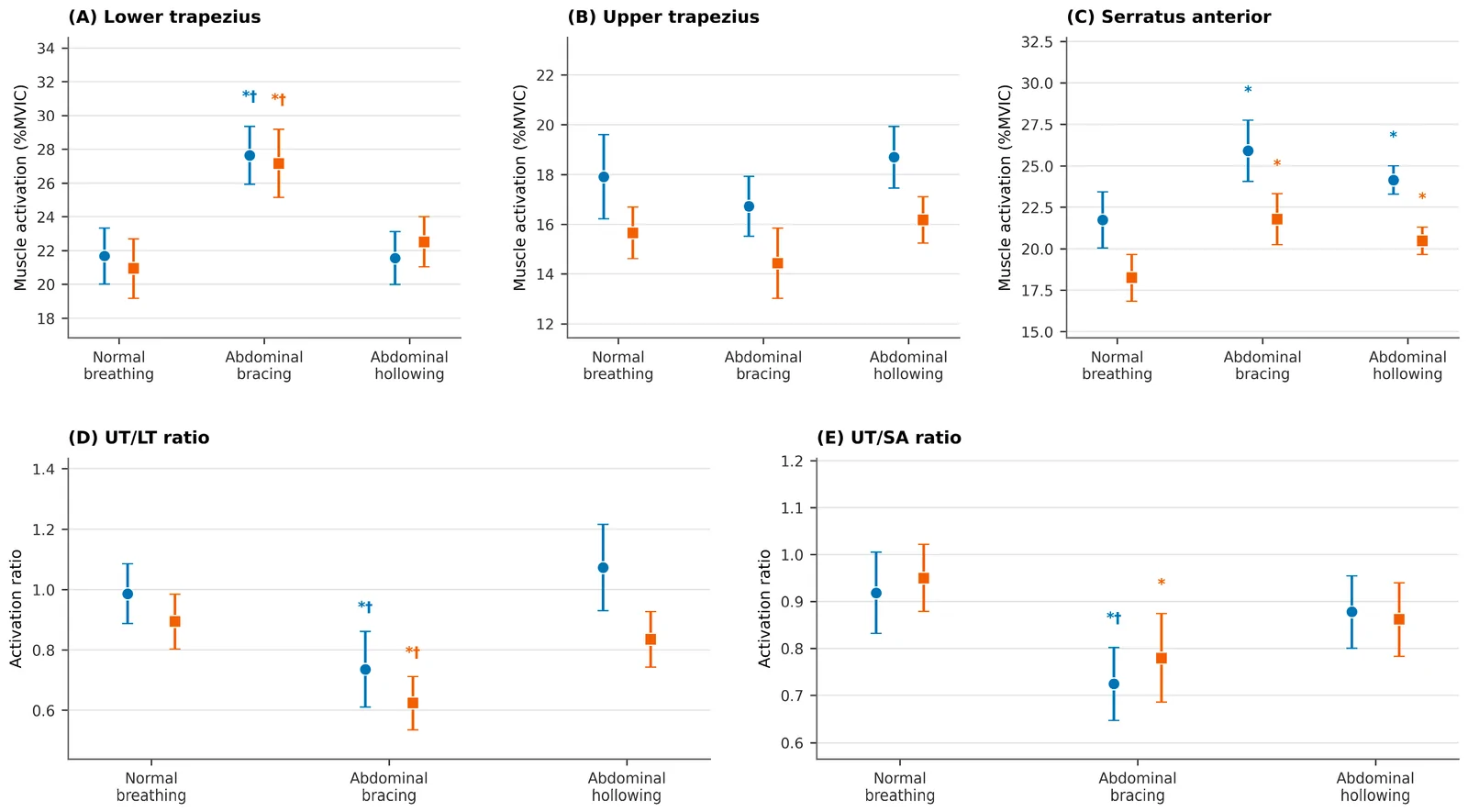
Scapular muscle activation and activation ratios across abdominal conditions during the Y-raise. (**A**) Lower trapezius activation, (**B**) upper trapezius activation, (**C**) serratus anterior activation, (**D**) UT/LT ratio, and (**E**) UT/SA ratio. Circles and squares represent the left and right sides, respectively. Abbreviations: LT, lower trapezius; UT, upper trapezius; SA, serratus anterior. Data are presented as means with within-subject 95% confidence intervals. * indicates a significant difference from normal breathing, and † indicates a significant difference from abdominal hollowing, based on Bonferroni-adjusted pairwise comparisons (*p* < 0.05).

Significant effects of abdominal condition were observed on scapular muscle activity during the Y-raise exercise (Table 2; Figure 1A–C). For the LT, a significant main effect was identified on both the left (*F* = 18.440, *p* < 0.001, $\eta_{p}^{2}=0.345$) and right sides (*F* = 13.803, *p* < 0.001, $\eta_{p}^{2}=0.283$). Bonferroni-adjusted pairwise comparisons showed that the bracing condition produced greater LT activation than both hollowing and normal breathing on both sides (*adjusted p* < 0.05), whereas hollowing and normal breathing did not differ (adjusted *p* > 0.05). UT activity did not differ significantly across conditions on either side (left: *F* = 2.094, *p* = 0.141, $\eta_{p}^{2}=0.056$; right: *F* = 2.519, *p* = 0.102, $\eta_{p}^{2}=0.067$). For the SA, a significant effect was observed on both sides (left: *F* = 7.823, *p* = 0.004, $\eta_{p}^{2}=0.183$; right: *F* = 7.915, *p* = 0.003, $\eta_{p}^{2}=0.184$). Pairwise comparisons showed that SA activation was higher in both the bracing and hollowing conditions than in normal breathing on both sides (adjusted *p* < 0.05), with no significant difference between bracing and hollowing (adjusted *p* > 0.05).

Muscle activation ratios also differed across conditions (Table 3; Figure 1D,E). Significant effects were observed for the UT/LT ratio on both the left (*F* = 8.220, *p* = 0.001, $\eta_{p}^{2}=0.190$) and right sides (*F* = 10.291, *p* < 0.001, $\eta_{p}^{2}=0.227$). The bracing condition yielded lower UT/LT ratios than both hollowing and normal breathing on both sides (adjusted *p* < 0.05), whereas hollowing and normal breathing did not differ (adjusted *p* > 0.05). Similarly, the UT/SA ratio varied significantly across conditions on both sides (left: *F* = 6.643, *p* = 0.002, $\eta_{p}^{2}$ = 0.160; right: *F* = 4.454, *p* = 0.015, $\eta_{p}^{2}$ = 0.113). On the left side, the bracing condition resulted in a lower UT/SA ratio than both normal breathing (adjusted *p* = 0.006) and hollowing (adjusted *p* = 0.016). On the right side, the bracing condition produced a lower UT/SA ratio than normal breathing (adjusted *p* = 0.021), whereas the other pairwise comparisons were not significant (adjusted *p* > 0.05).

Detailed results of the Bonferroni-adjusted pairwise comparisons are presented in Supplementary Table S1.

## 4. Discussion

The present study examined the acute effects of abdominal bracing, abdominal hollowing, and normal breathing on scapular muscle activation during the Y-raise exercise in young adults with RSP. The main finding was that abdominal bracing was associated with greater LT activation and lower UT/LT ratios compared with abdominal hollowing and normal breathing, whereas UT activity did not differ across conditions. In contrast, SA activation increased during both bracing and hollowing compared with normal breathing, with no significant difference between the two abdominal strategies. These findings suggest that abdominal bracing may acutely alter the relative activation balance of scapular muscles during the Y-raise, primarily through increased LT activation rather than suppression of UT activity.

One possible explanation for the greater LT activation observed during abdominal bracing is the distinct neuromuscular activation profile of this strategy. Unlike abdominal hollowing, which primarily emphasizes selective activation of deep local stabilizers, abdominal bracing involves co-contraction of both deep and superficial trunk muscles and has been associated with increased intra-abdominal pressure and greater global trunk stiffness [13,14]. In theory, this increased proximal stability may provide a more stable mechanical base for upper-limb movement and may facilitate force transmission between the trunk and shoulder complex [31]. However, trunk stiffness, intra-abdominal pressure, and abdominal muscle activity were not directly measured in the present study. Therefore, the role of proximal stiffness should be interpreted as a plausible mechanism rather than a confirmed explanation. The observed differences in scapular muscle activation may also have been influenced by differences in cueing, attentional focus, breathing control, or individual ability to perform the abdominal activation strategies. Although the same band resistance and anchor distance were used for all participants, resistance was not individualized or quantified; therefore, differences in relative exercise intensity may have contributed to variability in EMG amplitude.

The finding that LT activation was greater during abdominal bracing than during abdominal hollowing and normal breathing may be relevant because the LT contributes to scapular upward rotation and posterior tilt during arm elevation [8,9]. Together with the SA, the LT plays an important role in the scapular upward rotation force couple, which is necessary for efficient scapulothoracic mechanics during overhead movement [8,9]. In the present study, the increase in LT activation occurred without a significant change in UT activation, suggesting that bracing may have shifted the relative activation pattern toward greater LT contribution rather than reducing UT activity. This interpretation is important because insufficient LT activation and excessive UT dominance have been reported in relation to altered scapular kinematics, impingement-related symptoms, and scapular dyskinesis [7,20]. Nevertheless, because the present study did not include a non-RSP control group or symptomatic patients, the observed LT response should be interpreted as an acute EMG finding in young adults with RSP, rather than evidence of correction of a clinical muscle imbalance.

In contrast to LT, SA activation increased during both abdominal bracing and abdominal hollowing compared with normal breathing, with no significant difference between the two abdominal strategies. This finding suggests that SA activation may be responsive to the presence of an abdominal activation cue during the Y-raise. Previous studies have also suggested potential links between abdominal control, trunk stabilization, and scapular muscle activation [10,11,12]. However, the absence of a statistically significant difference between bracing and hollowing should not be interpreted as evidence that the two strategies have equivalent effects on SA activation, because equivalence testing was not performed. In addition, because abdominal muscle activation was not objectively verified, it remains unclear whether the SA response was related to abdominal muscle recruitment itself, breathing control, tactile cueing, or other task-related factors. Further studies using objective verification of abdominal activation are needed to clarify whether bracing and hollowing produce different effects on SA recruitment.

Beyond individual muscle activation, the present study demonstrated that UT/LT and UT/SA ratios were reduced under the bracing condition. These ratios provide information about the relative activation of scapular muscles during the Y-raise and have been used to examine scapular muscle activation patterns during functional shoulder exercises [30]. Elevated UT/LT and UT/SA ratios have been associated with scapular dyskinesis and shoulder dysfunction [32]. In the present study, the lower UT/LT ratio observed during bracing appears to be primarily attributable to increased LT activation rather than decreased UT activation. Therefore, the lower ratios should be interpreted as changes in relative EMG amplitude rather than as evidence of improved muscle balance, neuromuscular coordination, or a more optimal movement strategy. Because these ratios were calculated from time-averaged EMG amplitudes, they do not capture temporal aspects of coordination, such as activation onset, co-contraction timing, or intermuscular coherence. It should also be noted that middle trapezius activity was not measured in this study, and its potential contribution to scapular stabilization and activation balance could not be determined [9,33]. From a clinical perspective, these findings suggest that incorporating abdominal bracing into upper-limb exercises like the Y-raise can serve as an effective movement strategy to optimize scapulothoracic muscle recruitment. By enhancing LT and SA recruitment while maintaining stable UT activity, bracing provides a viable trunk-stabilization platform for clinicians aiming to restore optimal muscle activation balance during overhead shoulder rehabilitation.

The findings of this study are limited to acute EMG responses during the Y-raise. In young adults with RSP, abdominal bracing was associated with greater LT activation and altered scapular muscle activation ratios during this specific task. Because scapular kinematics, trunk stiffness, and abdominal muscle activity were not measured, the physiological mechanisms underlying these EMG differences remain uncertain. In addition, the participants were asymptomatic, and pain, functional performance, and rehabilitation outcomes were not assessed. Accordingly, abdominal bracing should be interpreted as an exercise cue that altered scapular muscle activation during a laboratory task rather than as a clinically effective rehabilitation strategy. Longitudinal clinical studies are needed to determine whether these acute EMG changes translate into meaningful functional or clinical benefits.

Several limitations of this study should be acknowledged. First, surface EMG is susceptible to crosstalk from adjacent muscles, particularly for the SA [34], and within-session reliability and measurement error were not formally quantified. In addition, MVIC normalization may have been influenced by participant effort and examiner-applied resistance, introducing variability into the %MVIC values and activation ratios. Second, correct performance of abdominal hollowing and bracing was verified during familiarization using clinical observation, palpation, and tactile feedback; however, abdominal activation was not objectively monitored during EMG data collection using abdominal EMG, ultrasound imaging, or pressure biofeedback. Therefore, the magnitude and consistency of the intended abdominal activation strategies throughout testing could not be confirmed and may have influenced the observed scapular EMG patterns. Third, only acute EMG responses during a single upper-limb task were examined; scapular kinematics, trunk stiffness, pain, and functional outcomes were not assessed. Thus, the findings cannot be generalized to other movements or interpreted as evidence of clinical improvement. Fourth, middle trapezius activity was not recorded, and its potential contribution to the observed changes in UT/LT ratios remains unquantified. Fifth, shoulder angle was measured once per side using smartphone-based photography, and intra-rater reliability was not assessed in the present study. Although the angle was used only for eligibility screening, marker placement, partial occlusion, lighting conditions, camera perspective, and image resolution may have introduced measurement error, as commonly reported for camera- and marker-based motion tracking systems [35,36]. Accordingly, RSP should be interpreted as a postural characteristic in asymptomatic adults rather than a clinical shoulder disorder. Finally, the sample was restricted to young adults with RSP, limiting generalizability to other age groups or clinical populations with diagnosed shoulder pathology. Future studies should incorporate objective verification of abdominal activation strategies, a broader range of tasks, more comprehensive muscle assessments, and clinically diagnosed populations to extend the applicability of these findings.

## 5. Conclusions

This study provides preliminary EMG evidence that, during the Y-raise, the abdominal bracing instruction was associated with greater LT activation and lower UT/LT ratios than hollowing and normal breathing, as well as lower UT/SA ratios than normal breathing, in asymptomatic young adults exhibiting RSP. These findings suggest that bracing instruction may acutely modify the relative scapular EMG activation pattern, primarily through greater LT activation rather than suppression of UT activity. Because abdominal activation and trunk stiffness were not objectively verified, the findings should be interpreted as responses to different instructional conditions. Further studies are needed to determine whether these acute EMG responses translate into improvements in scapular movement, pain, or function in symptomatic populations.

## Figures and Tables

**Table 1:** General characteristics of participants.

Variables	Participants (*n* = 36)
Age (years)	23.44 ± 2.36
Height (cm)	178.83 ± 5.55
Weight (kg)	73.58 ± 10.37
Left round shoulder angle (°)	37.40 ± 3.20
Right round shoulder angle (°)	39.70 ± 4.97

**Table 2:** Muscle activation during Y-raise with normal, bracing and hollowing conditions.

Muscle		Normal Breathing	Abdominal Bracing	Abdominal Hollowing	*p*	$\eta_{p}^{2}$	Post Hoc (Bonferroni)
Lower Trapezius	Left	21.67 ± 8.96	27.64 ± 9.72	21.55 ± 7.00	<0.001	0.345	B > H = N
	Right	20.93 ± 10.26	27.16 ± 12.77	22.51 ± 10.18	<0.001	0.283	B > H = N
Upper Trapezius	Left	17.91 ± 15.50	16.73 ± 11.52	18.70 ± 12.03	0.141	0.056	-
	Right	15.65 ± 8.13	16.18 ± 8.19	14.44 ± 9.30	0.102	0.067	-
Serratus Anterior	Left	21.73 ± 8.87	24.15 ± 9.02	25.91 ± 11.00	0.004	0.183	B = H > N
	Right	18.25 ± 6.70	21.78 ± 8.40	20.48 ± 6.83	0.003	0.184	B = H > N

Abbreviations: N, normal breathing; B, abdominal bracing; H, abdominal hollowing.

**Table 3:** Comparison of UT/LT and UT/SA ratios between bracing, hollowing, and normal conditions during Y-raise.

Muscle Ratio		Normal Breathing	Abdominal Bracing	Abdominal Hollowing	*p*	$\eta_{p}^{2}$	Post Hoc (Bonferroni)
UT/LT	Left	0.99 ± 0.96	0.74 ± 0.68	1.07 ± 1.12	0.001	0.190	B < H = N
	Right	0.89 ± 0.57	0.62 ± 0.54	0.83 ± 0.56	<0.001	0.227	B < H = N
UT/SA	Left	0.92 ± 0.67	0.72 ± 0.51	0.88 ± 0.63	0.002	0.160	B < H = N
	Right	0.95 ± 0.71	0.78 ± 0.85	0.86 ± 0.60	0.015	0.113	B < N; B = H; H = N

Abbreviations: N, normal breathing; B, abdominal bracing; H, abdominal hollowing.

## Data Availability

The data that support the findings of this study are available from the corresponding author upon reasonable request.

## Supplementary Materials

The following supporting information can be downloaded at: https://www.mdpi.com/article/10.3390/jcm15187282/s1, Table S1: Pairwise comparisons of scapular muscle activation and activation ratios across abdominal conditions.
