# Supplementary material for: Differential Effects of Abdominal Bracing and Hollowing on Scapular Muscle Activation Patterns During the Y-Raise Exercise
Source: J Clin Med. 2026 Sep 19;15(18):7282. doi: 10.3390/jcm15187282 (PMC13607870; doi:10.3390/jcm15187282)
Supplement: Supplementary file 1 [file jcm-15-07282-s001.zip › jcm-4471104-supplementary.pdf]

Table S1. Pairwise comparisons of scapular muscle activation and activation ratios across abdominal conditions

Outcome	Side	Comparison	Mean difference (Bonferroni-adjusted 95% CI)	Cohen's *dz* (95% CI)	Adjusted *p*
LT activation (%MVIC)	Left	Abdominal bracing − Normal breathing	5.97 (2.95 to 8.98)	0.83 (0.45 to 1.21)	<0.001
		Abdominal hollowing − Normal breathing	-0.12 (-2.90 to 2.66)	-0.02 (-0.35 to 0.31)	1.000
		Abdominal bracing − Abdominal hollowing	6.09 (3.24 to 8.94)	0.89 (0.50 to 1.28)	<0.001
	Right	Abdominal bracing − Normal breathing	6.23 (2.71 to 9.74)	0.74 (0.37 to 1.11)	<0.001
		Abdominal hollowing − Normal breathing	1.58 (-1.01 to 4.18)	0.26 (-0.08 to 0.59)	0.402
		Abdominal bracing − Abdominal hollowing	4.64 (1.53 to 7.75)	0.63 (0.26 to 0.98)	0.002
SA activation (%MVIC)	Left	Abdominal bracing − Normal breathing	4.18 (0.72 to 7.64)	0.51 (0.16 to 0.85)	0.014
		Abdominal hollowing − Normal breathing	2.42 (0.46 to 4.37)	0.52 (0.17 to 0.86)	0.011
		Abdominal bracing − Abdominal hollowing	1.76 (-0.59 to 4.12)	0.31 (-0.02 to 0.65)	0.204
	Right	Abdominal bracing − Normal breathing	3.53 (0.68 to 6.39)	0.52 (0.17 to 0.86)	0.011
		Abdominal hollowing − Normal breathing	2.23 (0.49 to 3.97)	0.54 (0.18 to 0.88)	0.008
		Abdominal bracing − Abdominal hollowing	1.30 (-0.73 to 3.34)	0.27 (-0.07 to 0.60)	0.349
UT/LT ratio	Left	Abdominal bracing − Normal breathing	-0.251 (-0.427 to -0.074)	-0.60 (-0.95 to -0.24)	0.003
		Abdominal hollowing − Normal breathing	0.087 (-0.129 to 0.302)	0.17 (-0.16 to 0.50)	0.955
		Abdominal bracing − Abdominal hollowing	-0.337 (-0.591 to -0.084)	-0.56 (-0.91 to -0.20)	0.006
	Right	Abdominal bracing − Normal breathing	-0.270 (-0.424 to -0.116)	-0.73 (-1.10 to -0.36)	<0.001
		Abdominal hollowing − Normal breathing	-0.059 (-0.221 to 0.103)	-0.15 (-0.48 to 0.18)	1.000
		Abdominal bracing − Abdominal hollowing	-0.211 (-0.367 to -0.055)	-0.57 (-0.92 to -0.21)	0.005
UT/SA ratio	Left	Abdominal bracing − Normal breathing	-0.194 (-0.341 to -0.047)	-0.55 (-0.90 to -0.20)	0.006
		Abdominal hollowing − Normal breathing	-0.041 (-0.187 to 0.105)	-0.12 (-0.44 to 0.21)	1.000
		Abdominal bracing − Abdominal hollowing	-0.153 (-0.283 to -0.024)	-0.50 (-0.84 to -0.15)	0.016
	Right	Abdominal bracing − Normal breathing	-0.170 (-0.320 to -0.021)	-0.48 (-0.82 to -0.13)	0.021
		Abdominal hollowing − Normal breathing	-0.088 (-0.207 to 0.030)	-0.31 (-0.65 to 0.02)	0.206
		Abdominal bracing − Abdominal hollowing	-0.082 (-0.242 to 0.078)	-0.22 (-0.54 to 0.12)	0.616

Note. Mean differences were calculated as the first condition minus the second condition.

Abbreviations: LT, lower trapezius; SA, serratus anterior; UT, upper trapezius; CI, confidence interval
